# Leiomyosarcoma of the Uterus: A Rare Diagnosis

**DOI:** 10.7759/cureus.17418

**Published:** 2021-08-24

**Authors:** Palak Paudel, Bibek Dhungana, Eliza Shrestha, Deepak Verma

**Affiliations:** 1 Gynaecology, Bhaktapur Cancer Hospital, Bhaktapur, NPL; 2 Internal Medicine, KIST Medical College, Lalitpur, NPL; 3 Internal Medicine/Family Medicine, California Institute of Behavioral Neurosciences & Psychology, Fairfield, USA

**Keywords:** hysterectomy, leiomyoma, uterus, neoplasm, menopause

## Abstract

Uterine leiomyosarcoma is a rare malignant tumor that accounts for almost 2-5% of all uterine malignancies. It has the highest prevalence during pre and perimenopause. Further, it clinically resembles benign conditions like leiomyoma, and the diagnosis is confirmed with the histologic findings of the mass. Here, we present the case of a 70-year-old female who presented with lower abdominal pain for two years. Exploratory laparotomy with hysterectomy was performed, and the diagnosis of leiomyosarcoma was confirmed after histological examination of the resected tumor.

## Introduction

Uterine leiomyosarcoma is a rare malignant tumor accounting for almost 2-5% of all uterine malignancies [1]. The highest prevalence is seen in the pre and perimenopausal age group [2]. The presenting symptoms are vague and often mimic other benign uterine conditions. Patients may present with abnormal uterine bleeding, pelvic pain, and/or uterine mass, although some patients remain asymptomatic. There is no reliable diagnostic method to distinguish between uterine leiomyosarcoma and benign uterine tumors before surgery. Hysterectomy is the treatment for early-stage disease, and the diagnosis is confirmed after histologic inspection of the resected tumor.

## Case presentation

A 70-year-old nulliparous woman presented to our hospital with complaints of lower abdominal pain in the hypogastric region, which was mild in nature and nonradiating for two years. She had her menopause 15 years back and did not have any known chronic illness. On examination, her vitals were stable; abdominal examination showed a fixed hard and nontender mass in the pelvic region of approximately 18-week size. On vaginal examination, her cervix appeared normal and the mass could not be felt separately.

She was referred to our center. Ultrasonography (USG) of the abdomen and endometrial biopsy were performed at another facility before presenting to our hospital. Endometrial biopsy showed mucoid tissue mixed with inflammatory cells only. Tumor markers including cancer antigen 125 (15.2 U/mL; normal: 0-35 U/mL) and carcinoembryonic antigen (1.27 ng/mL; normal: 0-2.5 ng/mL) were also tested earlier. A magnetic resonance imaging (MRI) of the abdomen and pelvis was advised, and exploratory laparotomy was planned because of the diagnostic dilemma. MRI showed a large heterogeneous signal lesion in the myometrium thinning the myometrial wall.

**Figure 1:**
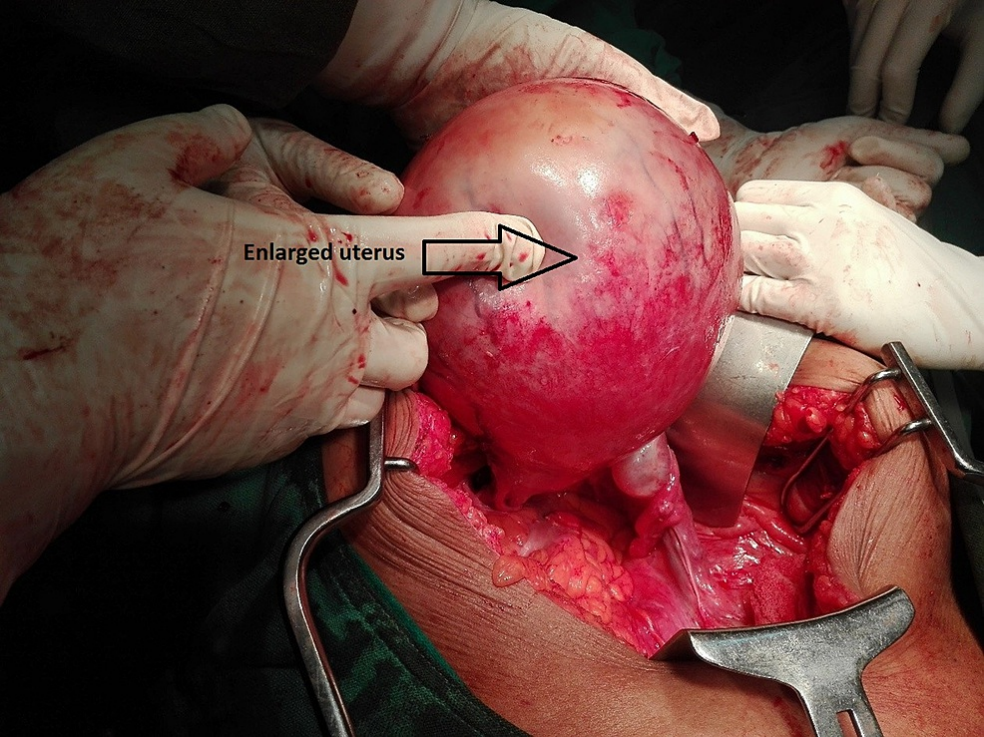
The enlarged uterus.

**Figure 2:**
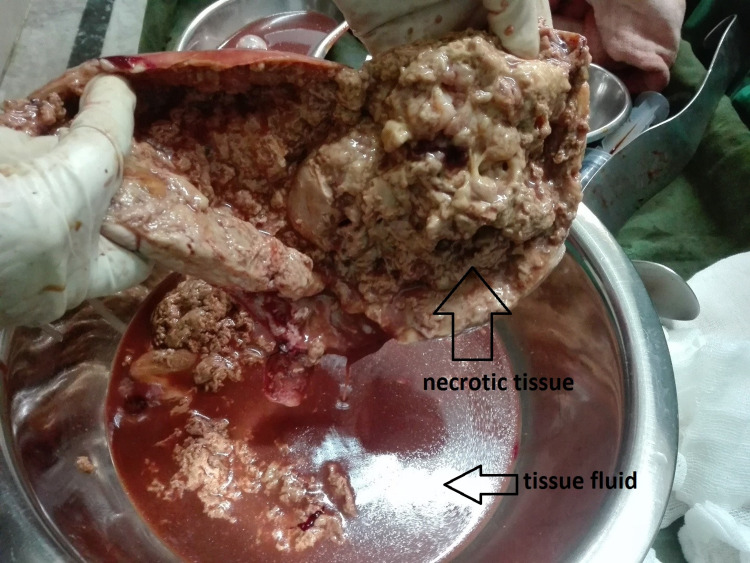
Cut section of the tumor showing necrotic tissue.

As the diagnosis could not be confirmed using MRI, we performed an exploratory laparotomy with a total abdominal hysterectomy and bilateral pelvic lymph node sampling with omentectomy. Operative findings were of a uterus at 24-week size (Figure 1), which was uniformly enlarged, and peritoneal fold densely adherent to the anterior wall of the uterus. Bilateral pelvic lymph nodes were enlarged with a maximum size of 1 × 1 cm. Bilateral tubes appeared normal and the omentum grossly looked healthy. There were no ascites. On the cut section (Figure 2), a mass of about 15 × 10 cm in size was seen arising from the fundus of the uterus containing abundant necrotic tissue and tissue fluid. The endometrial cavity looked normal.

**Figure 3:**
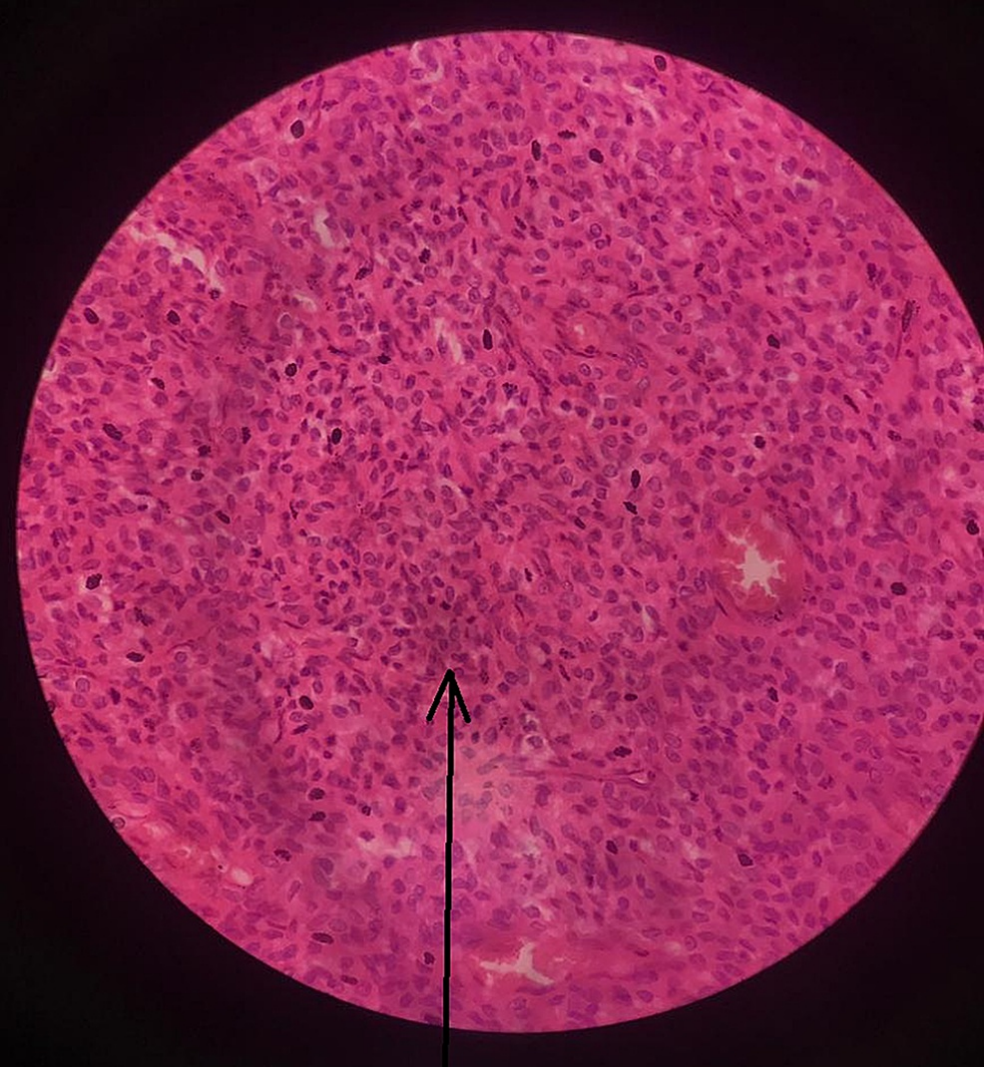
Tumor cells with indistinct cytoplasm.

**Figure 4:**
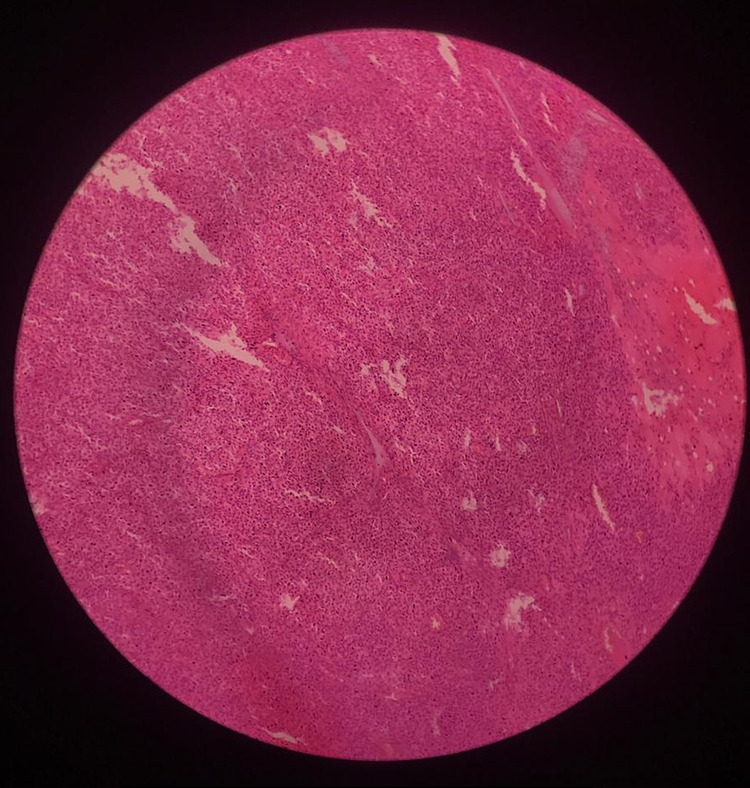
Tumor cells seen on low magnification (10×).

**Figure 5:**
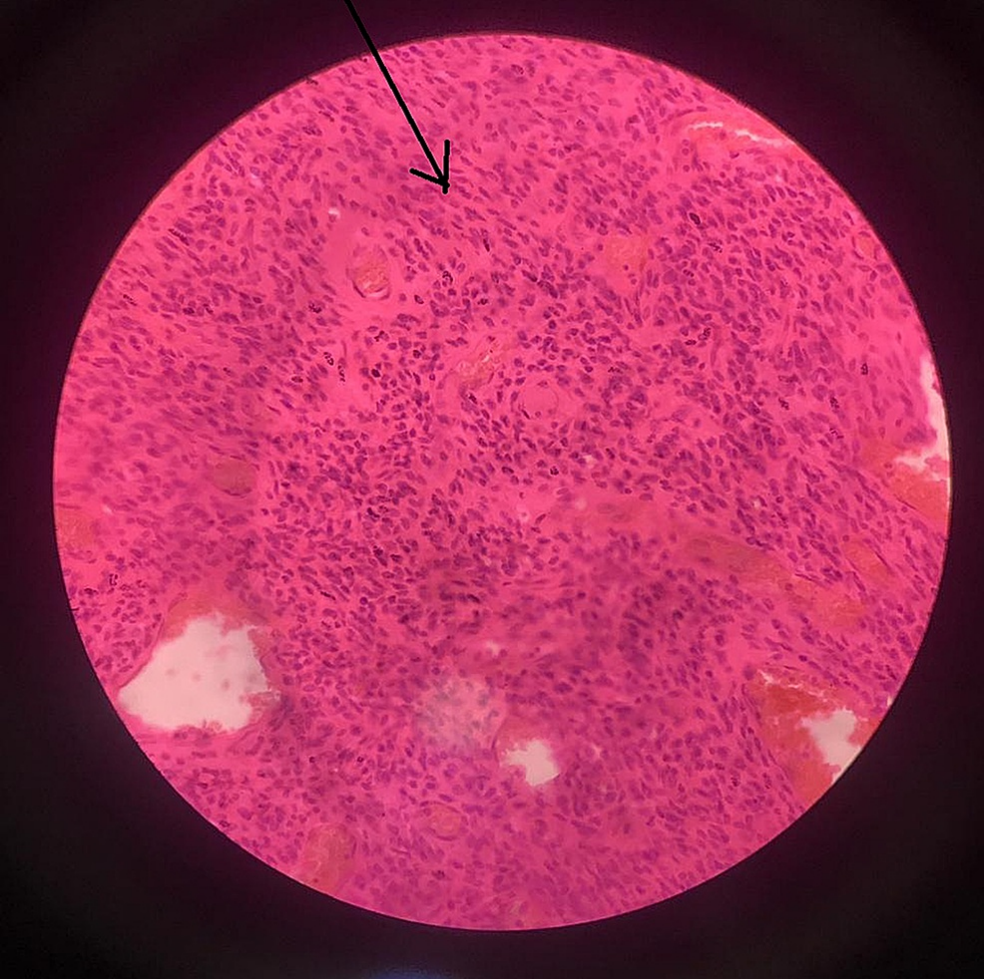
Fascicles of tumor cells seen on 40× magnification.

Histopathology examination of the mass (Figures 3-5) showed a large area of coagulative tumor necrosis.

The viable area showed fascicles of tumor cells with indistinct cytoplasm. Nuclei were oval to elongated and had coarse nuclear chromatin. Nuclear pleomorphism was moderate, and mitosis was noted. These findings were consistent with leiomyosarcoma. Moreover, the omentum was unremarkable, and the right and left external iliac lymph nodes were free of tumor. The histological examination of the tumor mass confirmed the diagnosis in our case.

## Discussion

Uterine leiomyosarcoma is a rare malignant tumor arising from the myometrium [1]. It accounts for almost 2-5% of all uterine malignancies, and six out of every 1,000,000 women in the United States are estimated to develop leiomyosarcoma each year [1]. The highest prevalence of uterine leiomyosarcoma is during pre and perimenopause with the average age of diagnosis being 51 years [2]. In our case, the patient was 70 years old. The pathogenesis of uterine leiomyosarcoma is not completely known; however, chromosomal rearrangement has been identified as an oncogenic mechanism by genome-wide studies conducted recently. Cell cycle regulators, p16 and p53, are frequently overexpressed and appear to be associated with the major modifications of the sarcomagenesis [3]. The risk factors for the development of de novo uterine leiomyosarcoma include obesity, menopausal use of estrogen and progestin, oral contraceptives, tamoxifen use, and nulliparity [4,5]. Our patient was a nulliparous woman. Furthermore, cigarette smoking has been associated with a reduced risk of developing leiomyosarcoma [5].

Uterine leiomyosarcoma can present with vague clinical symptoms and imitate benign conditions such as leiomyoma [6]. Patients may present with abnormal uterine bleeding, pelvic pain, and/or uterine mass, although some patients remain asymptomatic. Our patient presented with a lower abdominal mass of two-year duration. In women suspected of having symptomatic leiomyoma or leiomyosarcoma, USG is the preferred initial imaging, while MRI is an excellent modality for imaging the pelvis. Although MRI findings help differentiate leiomyoma from leiomyosarcoma, it is not a pathognomic imaging criterion because the currently available data are limited to demonstrate its utility [7]. Leiomyosarcoma is usually seen as a large infiltrating myometrial mass of heterogeneous hypointensity with ill-defined and irregular margins on T1-weighted MRI. Similar to T2-weighted MRI, leiomyosarcoma is seen as intermediate-to-high signal intensity with central hyperintensity, which is indicative of extensive necrosis [8].

The pathological diagnosis of leiomyosarcoma depends upon the presence of necrosis, mitosis, and cytologic atypia in the proliferating muscle cells [9]. According to the Stanford criteria, reported by Bell et al., the histologic diagnosis depends upon the presence of at least two of the following criteria: diffuse moderate-to-severe atypia, a mitotic count of at least 10 mitotic figures/10 HPF, and tumor cell necrosis [10]. Leiomyosarcoma is highly aggressive and has a very unfavorable prognosis. The age of the patient, race, and the International Federation of Gynecology and Obstetrics stage are the main prognostic factors along with the mitotic index and the hormonal receptor expression in the tumor [9]. Thus, the diagnosis of leiomyosarcoma before the age of 50 in an early stage with an absence of vascular invasion along with low myometrial invasion and histologic grade signifies a good prognosis [11].

Hysterectomy is the preferred treatment for early-stage disease along with complete resection of the gross tumor [7]. Systemic treatment and radiotherapy are of no proven value in the adjuvant setting [2]. Although there is no definite treatment for advanced and recurrent disease, significant progress has been made recently. Immunotherapies such as nivolumab and pembrolizumab along with novel chemotherapeutics such as olaratumab and pazopanib have been seen to work in advanced leiomyosarcoma [6].

## Conclusions

Uterine leiomyosarcoma is a rare malignant tumor with vague symptoms that often mimic other benign uterine conditions. It is difficult to differentiate uterine leiomyoma from leiomyosarcoma before surgery as the diagnostic tools are not reliable. Thus, the diagnosis is confirmed by the histopathological examination of the tumor mass done after surgery. Hysterectomy is the preferred treatment for early-stage disease.
